# Fe-capsaicin nanozyme attenuates sepsis-induced acute lung injury by regulating the functions of macrophages

**DOI:** 10.3389/fbioe.2024.1509136

**Published:** 2024-11-18

**Authors:** Ruijie Wang, Ning Zhou, Jinfang Xue, Yan Li, Yang Wang, Huadong Zhu, Chuanzhu Lv

**Affiliations:** ^1^ Emergency Department, The State Key Laboratory for Complex, Severe and Rare Diseases, Peking Union Medical College Hospital, Chinese Academy of Medical Science and Peking Union Medical College, Beijing, China; ^2^ Research Unit of Island Emergency Medicine, Chinese Academy of Medical Sciences (No. 2019RU013), Hainan Medical University, Haikou, China; ^3^ Emergency Department, Central People’s Hospital of Zhanjiang, Zhanjiang, Guangdong, China; ^4^ Emergency Medicine Center, Sichuan Provincial People’s Hospital, University of Electronic Science and Technology of China, Chengdu, China

**Keywords:** capsaicin, sepsis, macrophage, nanoparticles, acute lung injury

## Abstract

**Background:**

In sepsis, the lung is one of the worst affected organs, often leading to acute lung injury (ALI). More and more evidence suggests that macrophages are also involved in the pathogenesis of ALI. In our previous study, we successfully synthesized Iron-capsaicin-based nanoparticles (Fe-CAP NPs) and found that it could inhibit the secretion of inflammatory cytokines to alleviate ALI. Here, we further explore the anti-inflammatory mechanism of Fe-CAP NPs.

**Methods:**

Bone marrow-derived macrophages (BMDM) and C57 mice were divided into four groups: control group, lipopolysaccharide (LPS) group, CAP + LPS group and Fe-CAP + LPS group. Western blot and Immunofluorescence were used to detect the expression of macrophage phenotypic markers CD86 and CD206 in BMDM and lung tissue. Fluorescence microbeads, Transwell and ROS kit were used to detect the phagocytosis, migration and ROS clearing capability of BMDM. Western blot was used to detect the expression of JAK2/STAT3 pathway and apoptosis proteins in BMDM. TUNEL kit and H&E staining were used to evaluate apoptosis and pathological changes in lung tissue.

**Results:**

*In vitro*, CD86 expression was increased in LPS, but decreased after Fe-CAP pretreatment. CD206 expression was the opposite. Fe-CAP reduced phagocytosis, migration and scavenged ROS in LPS-treated BMDM. Fe-CAP inhibited P-JAK2 and P-STAT3 expression and reduced apoptosis. *In vivo*, Fe-CAP improved lung histopathology and reduced apoptosis in lung tissue of LPS group. CD86 expression was increased in lung tissue of LPS group, but decreased in Fe-CAP preconditioning, and CD206 expression was reversed.

**Conclusion:**

Fe-CAP NPs could alleviate sepsis-induced ALI by regulating the polarization and function of macrophages, reducing ROS level and apoptosis.

## Introduction

Sepsis is defined as a life-threatening organ dysfunction caused by a dysfunctional host response to infection (Poston and Koyner, 2019; van der Poll et al., 2017). A recent Global Burden of Disease study (2017) showed that sepsis is a significant global healthcare burden, with nearly 20% of reported deaths globally attributable to sepsis (Rudd et al., 2020). In sepsis, the lungs are particularly vulnerable to damage, often leading to acute lung injury (ALI) (Mayr et al., 2014). ALI is a series of clinical syndromes caused by the damage of alveolar epithelial cells and capillary endothelial cells, which is characterized by extensive damage to alveolar parenchyma and intractable hypoxemia (He et al., 2021). These pathological changes result in pulmonary edema, impaired oxygenation, and respiratory failure (Thompson et al., 2017). In severe cases, ALI can develop into acute respiratory distress syndrome (ARDS) (Ranieri et al., 2012). Because of its high morbidity, high mortality and limited clinical treatment, it has become the focus and difficulty of clinical work.

Capsaicin is a naturally occurring active compound found in chili peppers that has demonstrated antioxidant, analgesic, and anti-tumor effects (Rollyson et al., 2014; Merritt et al., 2022). However, its practical application is constrained by its hydrophobic property.

Nanozymes, a group of nanomaterials with enzyme-like properties, have garnered significant interest in the field of bio-medicine due to their remarkable stability and cost-effectiveness (Ren et al., 2022; Jiang et al., 2024; Lee et al., 2022). In addition, iron-based nanoenzymes have also been widely used due to their good properties (Sharma et al., 2023). In previous studies, we combined iron with the anti-inflammatory drug capsaicin, developed iron-capsaicin-based nanoparticles (Fe-CAP NPs), and investigated their potential to treat ALI. The results indicated that Fe-CAP NPs could decrease the expression of inflammatory cytokines in the lung via the nuclear factor κb (NF-κB) pathway (Wang et al., 2024). However, the specific mechanism of Fe-CAP alleviating ALI is still unclear and needs further study.

Macrophages, which originate from monocytes within the innate immune system, are ubiquitous in human tissues (Guo et al., 2022). They have a high degree of plasticity and function to regulate tissue homeostasis and adaptive responses. Activated macrophages are pathological markers of immune and inflammatory responses in ALI (Zhang et al., 2021; Fowler et al., 2019). In general, macrophages could differentiate into two main types: pro-inflammatory (M1) and anti-inflammatory (M2) macrophages (Sica et al., 2015). According to the report, exposed to lipopolysaccharide (LPS), the total number of macrophages increased significantly, mainly is the M1 macrophages and their corresponding expression products increase, and M2 macrophages and their corresponding expression products decrease (Murray et al., 2014). Conversely, the increase of M2-type macrophages can promote the repair and regeneration of alveoli and promote the recovery of ALI (Fan and Fan, 2018). Therefore, the balance of M1 and M2 macrophages and function is of great significance for the treatment of ALI.

In this study, bone marrow-derived macrophages (BMDM) and LPS-induced mouse sepsis models were used to demonstrate that Fe-CAP NPs alleviate septic lung injury by regulating macrophage polarization and function, reducing ROS level and apoptosis.

## Materials and methods

### Reagents and chemicals

The reagents used in the experiment were as follows: FeCl_3_⋅6H_2_O (aladdin, F102739-55 g), Capsaicin (MCE, 404864-50 mg), polyvinylpyrrolidone (PVP) (aladdin, P434440-250 g), lipopolysaccharide (LPS) (Sigma, L3129-10 MG), PBS (Gibco, 8123157), DMEM culture medium (Gibco, C11995500BT), Fetal bovine serum (Gibco, 30044333), M-CSF protein (MCE, HY-P7085), Color Pre-stained Protein Marker (Thermo, 26,619), Primary and secondary antibody dilution solution (BOSTER, 17K28C17), Pierce TM BCA protein Assay kit (Thermo Fisher Scientific, Prod # 23227), β-actin (Cell Signaling Technology, 4970S), Anti-Mannose Receptor (CD206) (abcam, ab125028), CD86 (Huabio, ET1606-50), JAK2 (Huabio, ET1607-35), p-JAK2 (Huabio, ET1607-34), STAT3 (Cell Signaling Technology, #12640), p-STAT3 (Cell Signaling Technology, #9145), Bcl-2 (Huabio, ET1602-53), Caspase (abcam, ab184787), Bax (Huabio, ET1603-34), F4/80 (Huabio, RT1212), anti-rabbit IgG, HRP-linked antibody (Cell Signaling Technology, 7074S), anti-mouse IgG, HRP-linked antibody (Cell Signaling Technology, 7076S), Secondary antibody (Thermo Fisher Scientific, Prod # A-21202), Secondary antibody (Thermo Fisher Scientific, Prod # A-31572), Reactive Oxygen Species Assay Kit (ROS, Beyotime, S0033S), SuleLumia ECL kit (Abbkine, K22020), Latex beads (Sigma, L3030), Calcein-AM Reagent (Dojindo, PJ671), Transwell permeable supports (Costar, United States).

### Isolation of BMDM

Bone Marrow-Derived Macrophages (BMDM) were isolated from the femur and tibia of male C57BL/6 mice. The procedure was as follows: Flushed the marrow cavity with DMEM medium containing 10% FBS and added red cell lysate to it. Subsequently, the primary macrophages were cultured in 25 cm^2^ bottles with 20 ng/mL macrophage colony-stimulating factor (100 ng/mL, abcam, United Kingdom, ab129146) for 7 days.

### Cell culture

The BMDM were cultured in Dulbecco’s Modified Eagle Medium (DMEM, Gibco by Thermo Fisher Scientific, United States, C11995500BT) supplemented with 10% fetal bovine serum (FBS, Gibco by Thermo Fisher Scientific, United States, 30044333). Then the cells were cultured in 25 cm^2^ bottles at 37°C in humidified containing 5% CO_2_ and 95% air. Every 48 h to replace part of the culture medium.

### Cell viability assay

Cell Counting Kit-8 (CCK-8, Beyotime, C0038) was used to assess the viability of BMDM cells. The BMDM cells (2 × 10^4^ cells/well) were inoculated into a 96-well plate. The cells were treated with different concentrations of CAP (capsaicin, MCE, United States) (ranging from 25 to 100 µM) and Fe-CAP (ranging from 7.5 to 30 μg/mL) and cultured for 0, 3, 6, and 9 h. After removing the old medium, 90 µL of fresh base medium and 10 µL of CCK-8 solution were added to per well. The cells were further incubated for 2 h, and the optical density was read at 450 nm using a microplate reader. To visually assess the effect of the above drug treatment, 5 µM calcein AM and 5 µM PI were added for 30 min and observed under a microscope. This experiment was repeated thrice.

### BMDM groups and treatments

BMDM cells were randomly divided into four groups: control group, LPS group, CAP + LPS group and Fe-CAP + LPS group. In the control group, the cells were cultured with basic medium (DMEM) for 8 h. In the LPS group, the cells were treated with LPS (1 μg/mL) for 6 h. In the CAP + LPS group, the cells were pretreated with CAP (50 µM) for 2 h, and then treated with LPS (1 μg/mL) for 6 h. In the Fe-CAP + LPS group, the cells were pretreated with Fe-CAP (15 μg/mL) for 2 h, and then treated with LPS (1 μg/mL) for 6 h. The cells were collected for protein extraction and immunofluorescence. The experiment was repeated three times.

### RNA sequencing

BMDM cells were treated based on the aforementioned groups. Subsequently, RNA extraction (Invitrogen TRIzol), purification (Thermo Scientific NanoDrop 2000; Thermo Scientific, Waltham, Massachusetts, United States), and library preparation (NEBNext Ultra II RNA library Prep Kit for Illumina) were conducted. The samples were sequenced using next-generation sequencing (NGS) on the Illumina platform and analyzed through RNA-seq using PANOMIX. The differential expression analysis of the two groups was performed using DESeq software (version 1.20.0). Gene Ontology (GO) enrichment analysis was carried out using topGO, with a significance level set at *P* < 0.05. Enrichment analysis of Kyoto Encyclopedia of Genes and Genomes (KEGG) pathways was executed using clusterProfiler software (version 3.4.4), focusing on pathways with a *P*-value <0.05. Differential splicing events were analyzed using rMATS software (version 3.2.5), concentrating on the five primary alternative splicing event types: SE, RI, MXE, A5SS, and A3SS.

### Phagocytosis assay

After the cells were treated according to the above group, they were incubated with red Latex beads (Sigma-Aldrich, L3030) for 1 h. The fluorescent microspheres that were not engulfed were removed by washing with PBS three times. Then it was fixed with 4% paraformaldehyde and washed with PBS thrice. The images were captured at 200× magnification. The experiment was carried out at least three times.

### ROS assay

BMDM were treated as described above. ROS levels in BMDM were detected using the Reactive Oxygen Species Assay Kit (ROS, Beyotime, S0033S). The specific steps were carried out according to the instructions. Fluorescence intensity analysis was performed using ImageJ software. The experiment was repeated three times.

### Cell migration assay

BMDM were seeded in a 24-well plate. Transwell chambers (Costar, United States) with an 8.0 µm pore size were used to evaluate the effect of drug on BMDM migration. First, 200 μL cell suspension (10^4^ cells) was added into the insert. Second, the cells were treated as described earlier. Third, 200 μL of serum-free medium was added to the upper cavity, and 500 μL of complete medium was added to the lower cavity. The cells were then incubated in an incubator at 37°C and 5% CO_2_ for 24 h. The old medium was discarded, and the inserts were carefully removed. The cells were then fixed with methanol and stained with 0.5% crystal violet. Cells that did not cross the membrane were gently erased with a wet cotton swab. The migrated cells were randomly captured under the microscope and counted using ImageJ software. The experiment was carried out at least three times.

### Ethics of animals

The use and handling of mice used for sepsis complied with the ethical guidelines outlined in the National Institutes of Health Guidelines for the Care and Use of Laboratory Animals. The use and experimental program of animals were approved by the ethics committee at No. 326, 2023 of Sichuan Provincial People’s Hospital. Every possible measure was taken to minimize the number of mice used and alleviate any potential suffering.

### Animals and treatments

Male C57 mice aged 6–8 weeks and weighing 18–22 g (Table 1) were procured from Byrness Weil biotech Ltd. Corporation. The mice received a standard laboratory diet and access to free water. The mice were randomly divided into four groups: sham, LPS (10 mg/kg, i. p.), CAP (8 mg/kg, i. p.) + LPS (10 mg/kg, i. p.) and Fe-CAP (8 mg/kg, i. p.) + LPS (10 mg/kg, i. p.). In the CAP + LPS group, mice were injected with CAP (8 mg/kg, i. p.) every 24 h followed by LPS at 96 h. Similarly, in the Fe-CAP + LPS group, mice received Fe-CAP (8 mg/kg, ig) every 24 h prior to LPS injection at 96 h. The mice were euthanized 12 h after LPS injection.

**TABLE 1:** Number of animals used in various treatments.

Usage		CON	LPS	CAP + LPS	Fe-CAP+LPS
Mouse	isolation of BMDM	N = 20			
	Lung pathology, WB	N = 3	N = 6 (1)	N = 6	N = 6
	Immunohistochemistry, TUNEL	N = 3	N = 3	N = 3	N = 3
Total		N = 26	N = 9 (1)	N = 9	N = 9

### Western blotting

BMDM were treated as described above. The left lung from mice was stored in liquid nitrogen at −80°C. Lysis buffer containing phosphatase and protease inhibitors (Pierce, Thermo Fisher Scientific Corporation, Bloomingdale, Illinois, United States) was added to the lung tissue and cells. The cell fragments from different groups were collected by scraping with a rubber spatula and centrifuged at 15,000 rpm for 15 min to collect the supernatant. The lung tissue were homogenized in an animal tissue grinder at 4°C and 80 Hz for 10 min, and the supernatant was collected. Protein concentration in cells and tissues was detected with the BCA protein detection kit (BCA, Beyotime, P0010). The protein sample were heated to 100°C for 10 min and stored at −80°C.

SDS-PAGE was used to separate proteins with different molecular weights. The isolated proteins were then transferred to a polyvinylidene fluoride (PVDF) membrane. The membrane was blocked with 5% milk at room temperature for 1 h. Subsequently, the membrane was incubated with primary antibody (1:1,000) and secondary antibody (1:5,000) overnight at 4°C and for 1 h at room temperature, respectively. SuleLumia ECL kit (Abbkine, United States, K22020) was employed for imaging. Band intensity was quantified using ImageJ software. Specific details of the antibodies used were shown in Table 2. The experiment was carried out at least three times.

**TABLE 2:** Antibodies used for Western blotting and immunofluorescence.

Antibody	Host	Source	Catalog number	Dilution for staining	Dilution for Western blot
CD86	Rabbit polyclonal	Huabio, China	ET1702-04	1:200	1:500
anti-Mannose	Rabbit polyclonal	Abcam, United Kingdom	ab125028		1:1,000
P-JAK2	Rabbit polyclonal	Huabio, China	ET1607-34		1:500
JAK2	Rabbit polyclonal	Huabio, China	ET1607-35		1:500
STAT3	Rabbit polyclonal	Cell Signaling Technology, United States	12,640		1:1,000
P-STAT3	Rabbit polyclonal	Cell Signaling Technology, United States	9,145		1:1,000
Bcl-2	Rabbit polyclonal	Huabio, China	ET1602-53		1:500
BAX	Rabbit polyclonal	Huabio, China	ET1603-34		1:10,000
Caspase	Rabbit polyclonal	Abcam, United Kingdom	ab184787		1:1,000
β-actin	Rabbit polyclonal	Cell Signaling Technology, United States	4970S		1:1,000
IgG-HRP	Mouse monoclonal	Thermo Fisher Scientific, United States	7076S		1:1,000
IgG-HRP	Rabbit polyclonal	Thermo Fisher Scientific, United States	7074S		1:1,000
Secondary Antibody	Donkey polyclonal	Thermo Fisher Scientific, United States	A-31572	1:200	
Secondary Antibody	Donkey polyclonal	Thermo Fisher Scientific, United States	A-21202	1:200	
F4/80	Rabbit polyclonal	Huabio, China	RT1212	1:200	

### Double immunofluorescence

In brief, BMDM were treated as described previously. First, BMDM cells were fixed in 4% paraformaldehyde at room temperature for 15 min. Second, it was blocked with 10% donkey serum for 1 h and washed with 1 × PBS three times for 5 min each time. Third, the cells were incubated with the corresponding primary and secondary antibodies at 4°C overnight and at room temperature for 1 h, respectively. DAPI was added and incubated for 10 min. Then, the cells were washed with 1 × PBS three times. The images were taken under a microscope at 200× magnification. The experiment was carried out at least three times.

The right lung of mice was used for double immunofluorescence. Twelve hours after injection of LPS, mice were anesthetized with 3% pentobarbital sodium (30 mg/kg), then infused with 2% paraformaldehyde and killed. After gradient dehydration, the lung tissue was embedded with OCT and sectioned on a 4 µm thickness microtome (Model: 2,165; Leica, Bensheim, Germany). Sections were then incubated with the corresponding primary and secondary antibodies at 4°C overnight and at room temperature for 1 h. Add DAPI to the slices and incubated for 10 min before washing with PBS for three times. The images were taken under a microscope with a magnification of 400×. The experiment was carried out at least three times.

### Histological examination and lung injury score (LIS)

Following 12 h of LPS injection, mice were anesthetized with 3% pentobarbital sodium (30 mg/kg). The right lung tissues were carefully excised, fixed with 4% paraformaldehyde, paraffin embedded, and sectioned at a thickness of 4 µm. The sections were stained conventionally with hematoxylin-eosin (H&E). Lung injury score (LIS) was quantified by blind method.

### TUNEL staining

ApopTag^®^ *In Situ* Apoptosis assay Kit was used to assess apoptosis. The kit used dUTP nickel end labeling (TUNEL) staining mediated by terminal deoxynucleotide transferase (TdT). The tissue sections were then observed under a fluorescence microscope (Olympus Corporation). This method can accurately detect and evaluate apoptosis in tissue samples. ImageJ soft was used to analyse the result.

### Statistical analysis

Statistical analysis was performed using GraphPad Prism 8.0 Software (GraphPad Software, San Diego, CA). After homogeneity of variance assessment, one-way analysis of variance (ANOVA) followed by Tukey test for multivariate comparison was used to determine the statistical significance of the differences between groups. *P* < 0.05 was considered statistically significant.

## Results

### Effects of CAP and Fe-CAP on viability of BMDM

**FIGURE 1:**
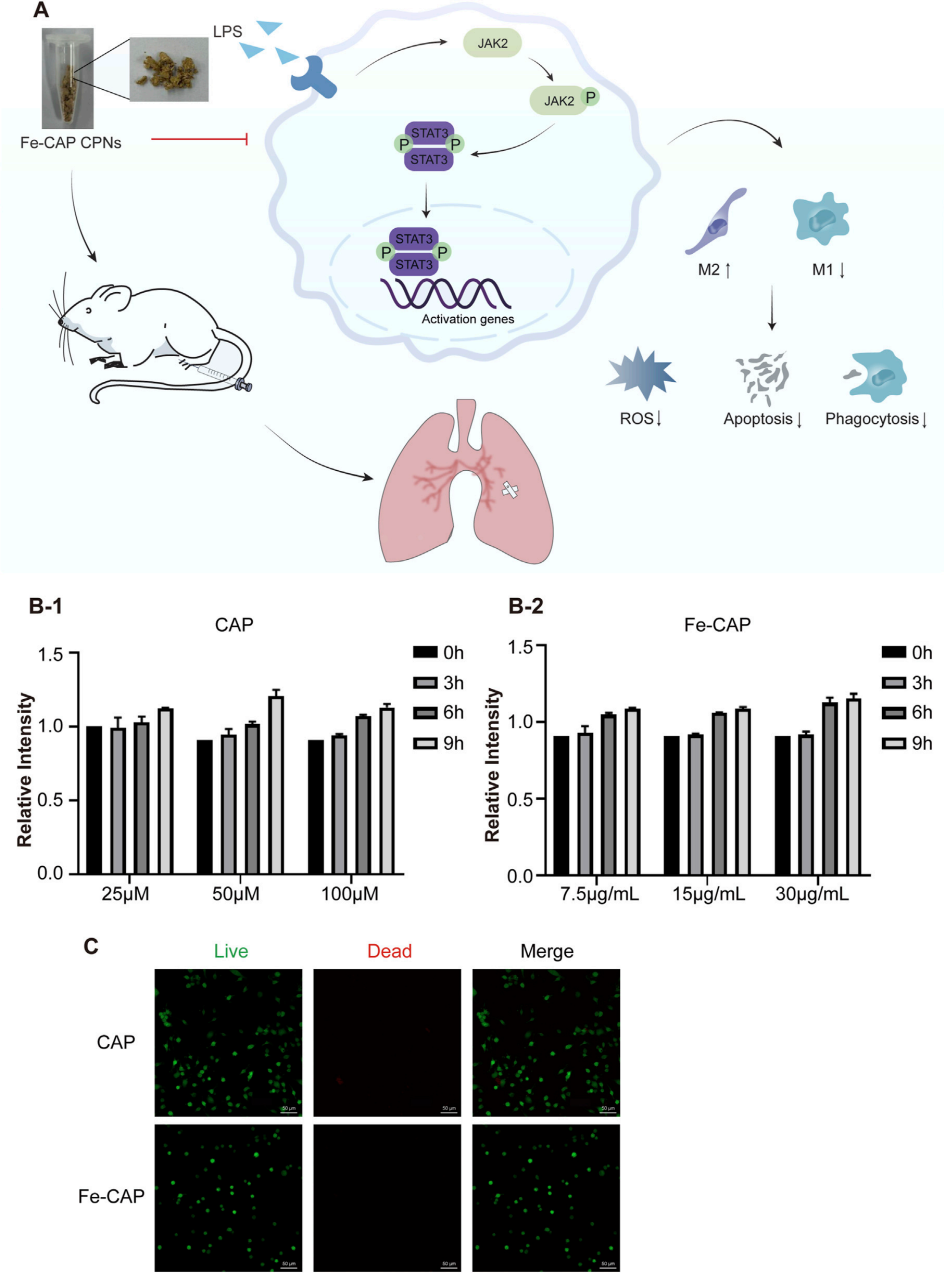
Effect of CAP and Fe-CAP NPs and LPS on viability of BMDM. Schematic illustration of the experiment conducted here **(A)**. In the range of 25–100 μM, CAP **(B-1)** did not affect the cell viability in 9 h. Fe-CAP **(B-2)** at the concentration between 7.5 μg/mL to 30 μg/mL did not affect the viability of BMDM in 9 h. The cytotoxicity of different treatments was observed by microscope live/dead cell staining **(C)**.

**FIGURE 2:**
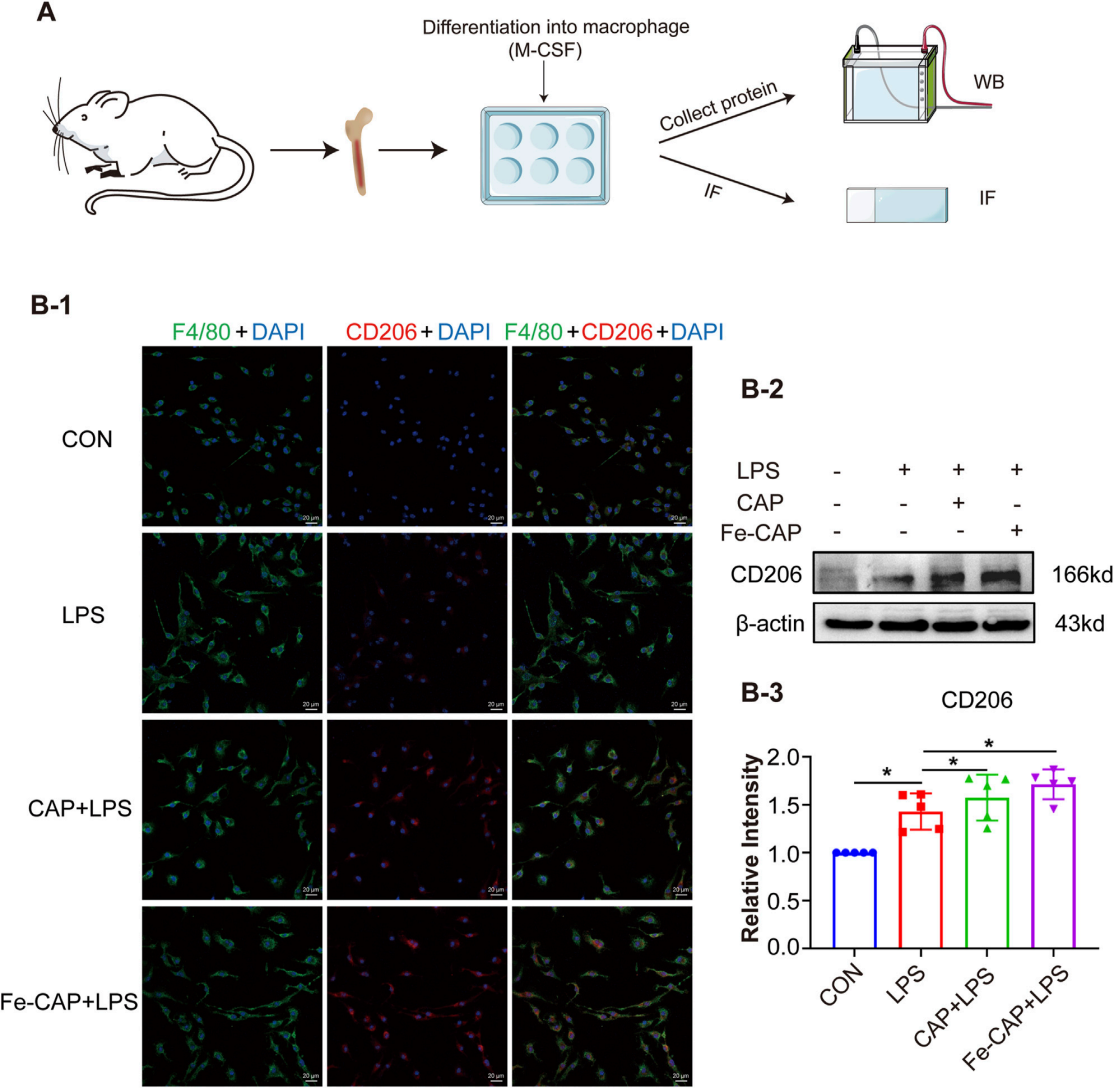
Fe-CAP NPs increased the polarization of BMDM towards M2 type. Schematic illustration of the experiment conducted here **(A)**. The expression of CD206 was detected by immunofluorescence **(B-1)** and Western blot **(B-2, B-3)**.

The flow chart of the whole experiment was shown in Figure 1A. CCK-8 assay was used to detect the cytotoxicity of CAP and Fe-CAP on BMDM cells. BMDM were exposed to concentrations ranging from 25 to 100 µM of CAP (Figure 1B–1) and 7.5–30 μg/mL of Fe-CAP (Figure 1B–2) for 9 h. The results indicated no significant alteration in cell viability following the treatments within the 9-h. Additionally, the cytotoxic effects of the various treatments were observed by live/dead cell staining under a microscope (Figure 1C), and the results showed that the cells in both CAP and Fe-CAP groups appeared green, which was consistent with the results of CCK-8 assay. Subsequently, we performed *in vitro* assays using CAP (50 µM) or Fe-CAP (15 μg/mL) for 8 h, and LPS (1 μg/mL) for 6 h, respectively.

### Fe-CAP NPs increased M2 macrophage polarization *in vivo*

The flow chart of the cell experiment was shown in Figure 2A. We investigated phenotypic markers of macrophage by Western blotting assay and immunofluorescence methods.

**FIGURE 3:**
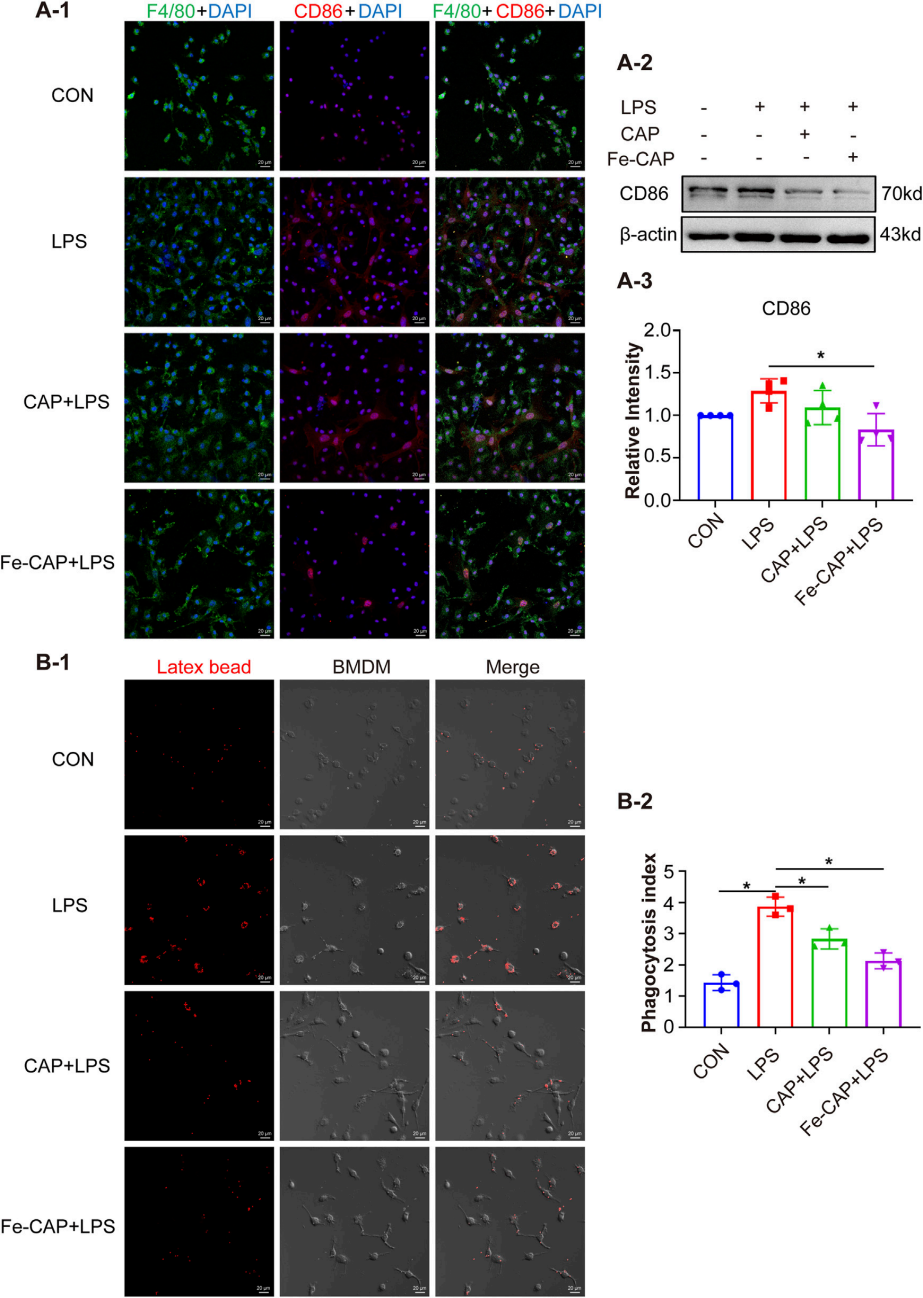
Fe-CAP NPs inhibits phagocytosis and polarization of BMDM to M1 type. The expression of CD86 was detected by immunofluorescence **(A-1)** and Western Blot **(A-2, A-3)**. Latex beads were used to detect the phagocytosis of BMDM **(B-1, B-2)**.

CD206 and CD86 are phenotypic markers of M2 macrophages and M1 macrophages, respectively. Western Blot results showed that the expression of CD206 (Figure 2B–2, 2B–3) in BMDM of Fe-CAP NPs + LPS group notably increased compared with LPS group. Interestingly, capsaicin alone also increased CD206 expression, but not as effectively as Fe-CAP NPs. Similarly, the expression of CD86 (Figure 3A–2) was increased in LPS group and significantly decreased after Fe-CAP pretreatment.

Immunofluorescence results demonstrated that Fe-CAP NPs effectively increased the fluorescence intensity of CD206 (Figure 2B–1) and decreased the fluorescence intensity of CD86 (Figure 3A–1). The above results indicated that Fe-CAP NPs could promote BMDM polarization to M2 type and inhibit BMDM polarization to M1 type after LPS treatment.

### Fe-CAP NPs decreased phagocytosis and migration of LPS-treated BMDM

Compared to the control, phagocytosis (Figure 3B) of BMDM in LPS group was increased, while that in Fe-CAP + LPS group was decreased.

**FIGURE 4:**
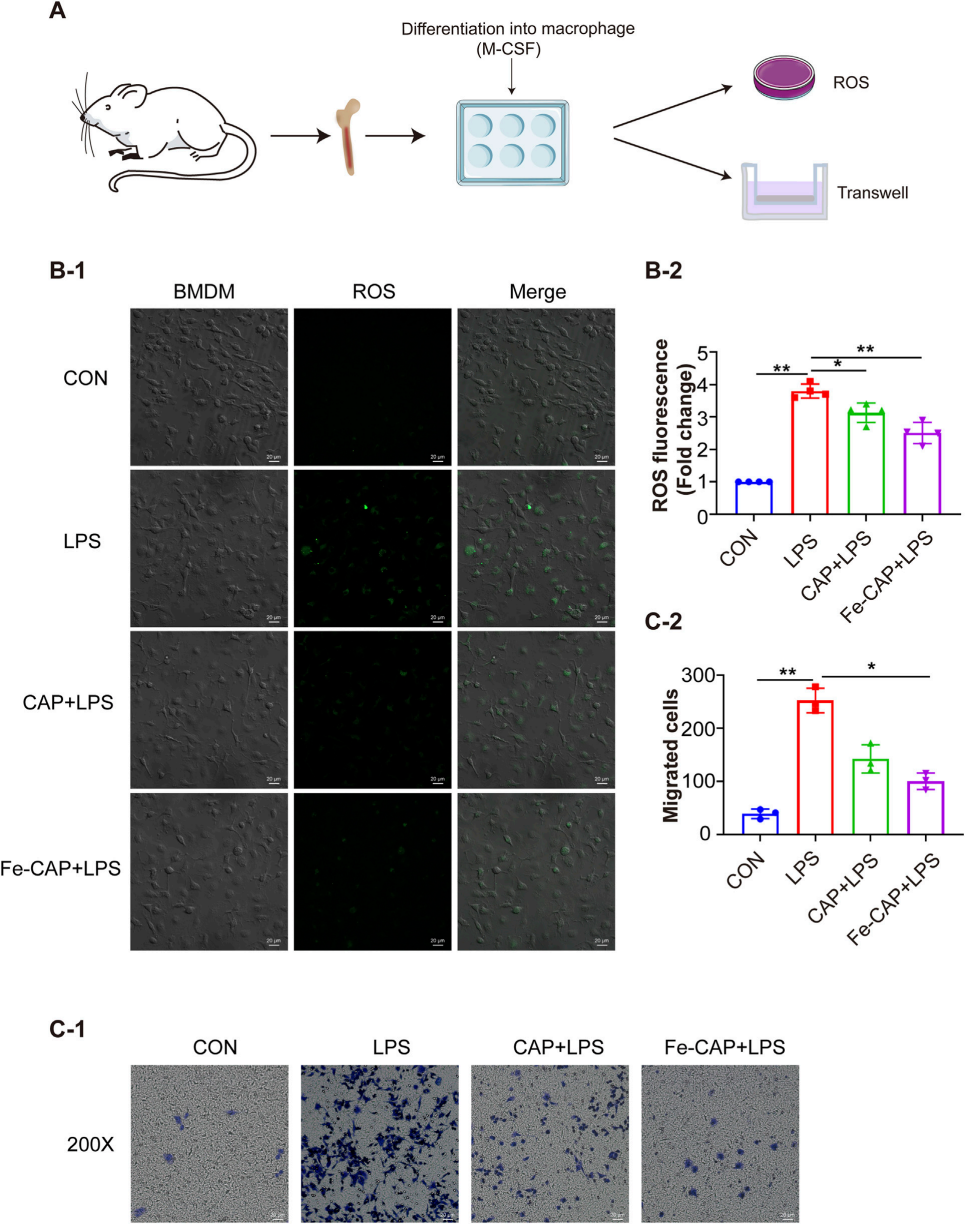
Fe-CAP NPs inhibited the ROS level and the migration of BMDM. Schematic illustration of the experiment conducted here **(A)**. The ROS levels were evaluated by DCFH-DA staining **(B-1, B-2)**. Transwell was used to detect the migration ability of macrophages **(C-1, C-2)**.

Transwell migration assay (Figure 4C) showed that compared with the control group, the migration of BMDM in the LPS group was notably increased, while the migration of macrophages in the Fe-CAP + LPS group was significantly decreased.

### Fe-CAP NPs pretreatment inhibited activation of JAK2/STAT3

**FIGURE 5:**
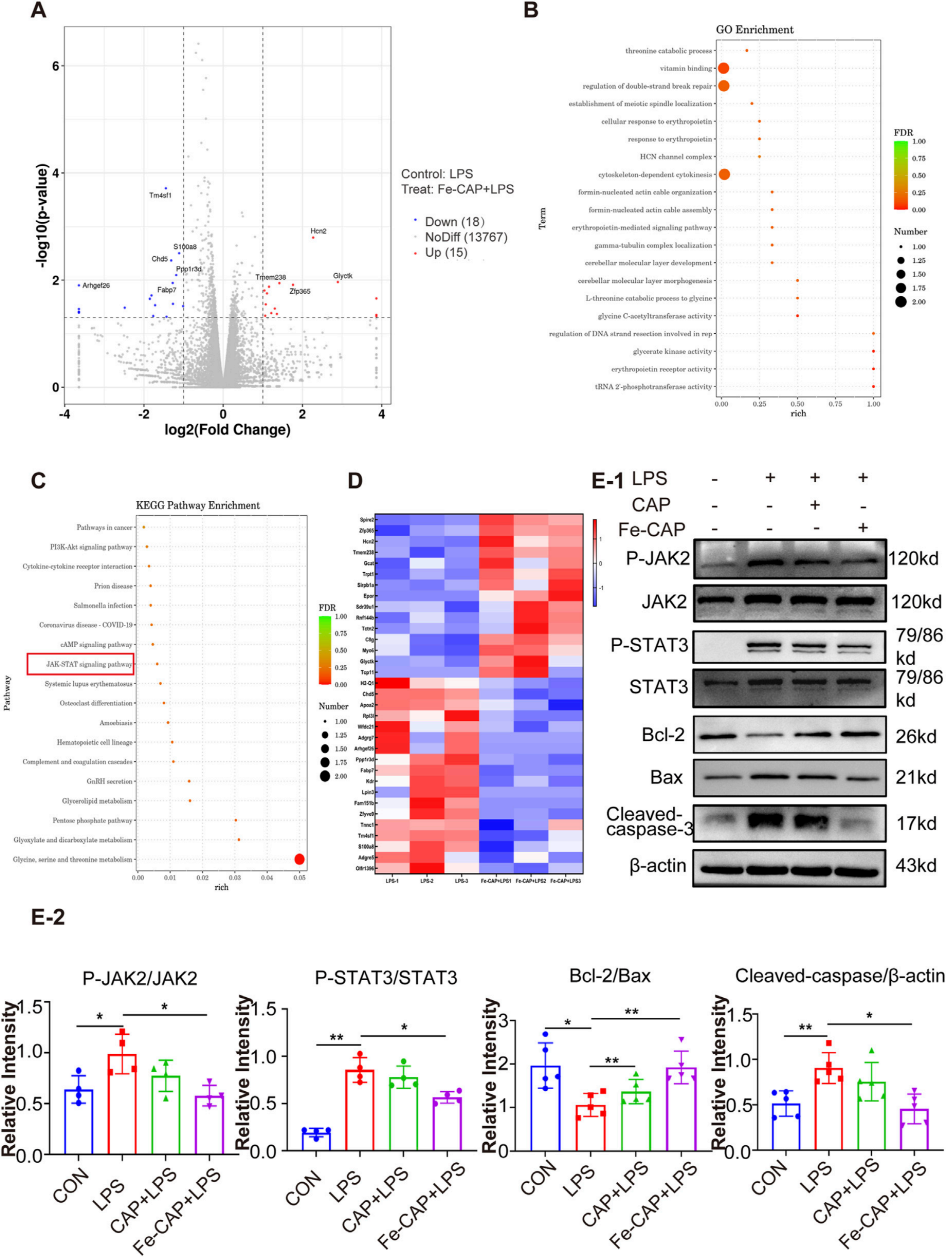
Fe-CAP reduced the apoptosis of macrophages. Volcano plot **(A)**. GO enrichment analysis of Fe-CAP pretreated BMDM after LPS stimulation (top 20 pathways) **(B)**. KEGG enrichment analysis of Fe-CAP pretreated BMDM after LPS stimulation (top 18 pathways) **(C)**. Heat map analysis of Fe-CAP pretreated BMDM after LPS stimulation **(D)**. P-JAK2, JAK2, P-STAT3 and STAT3 levels were detected by Western blot **(E)**. The expression levels of Bcl-2/BAX/cleaved caspase-3 proteins in BMDM cells were evaluated by Western blot **(E-1, E-2)**.

To further understand how Fe-CAP ameliorated ALI, RNA sequencing was conducted using the Illumina platform to investigate the pathway of Fe-CAP on LPS-treated macrophages polarization. The RNA sequencing results shown in the figure were all obtained from a comparison between the LPS group and the Fe-CAP + LPS group. The volcano plot (Figure 5A) indicated that 15 genes were upregulated and 18 genes were downregulated. GO (Figure 5B) and KEGG (Figure 5C) enrichment analyses were used to identify pathways associated with macrophage polarization. A heat map was plotted, and cluster analyses were performed (Figure 5D). The results suggested that inhibition of JAK2/STAT3 activation may be one of the mechanisms by which Fe-CAP regulated the polarization of macrophages.

Therefore, we used Western Blot to detect the activation of JAK2 and STAT3. The results demonstrated that LPS stimulation led to significant increase in the phosphorylated forms of JAK2 (Figures 5E–1, 5E–2) and STAT3 (Figures 5E–1, 5E–2). However, pretreatment with Fe-CAP partially reversed these protein changes, showing a more pronounced effect compared to CAP alone. These results indicated that pretreatment with Fe-CAP could promote M2 macrophage polarization by inhibiting the activation of JAK2 and STAT3 signaling pathways.

### Fe-CAP NPs scavenged ROS and inhibited apoptosis in LPS-treated BMDM cells

The flow chart of the cell experiment was shown in Figure 4A. The results indicated that macrophages showed higher fluorescence after LPS treatment. The effect of scavenging ROS (Figure 4B) with CAP alone was not obvious. However, Fe-CAP NPs were effective in scavenging ROS.

Western Blot was used to assess apoptosis of macrophages in different groups. The results showed that the expression of proapoptotic protein Bax (Figures 5E–1, 5E–2) and Cleaved caspase-3 (Figures 5E–1, 5E–2) in BMDM significantly increased in the LPS group, while decreased in Fe-CAP NPs + LPS group. Conversely, the expression of anti-apoptosis protein Bcl-2 (Figures 5E–1, 5E–2) decreased in LPS group, which was reversed by pretreatment with Fe-CAP NPs.

### Fe-CAP NPs alleviated histopathological changes and reduced apoptosis of lung tissue

**FIGURE 6:**
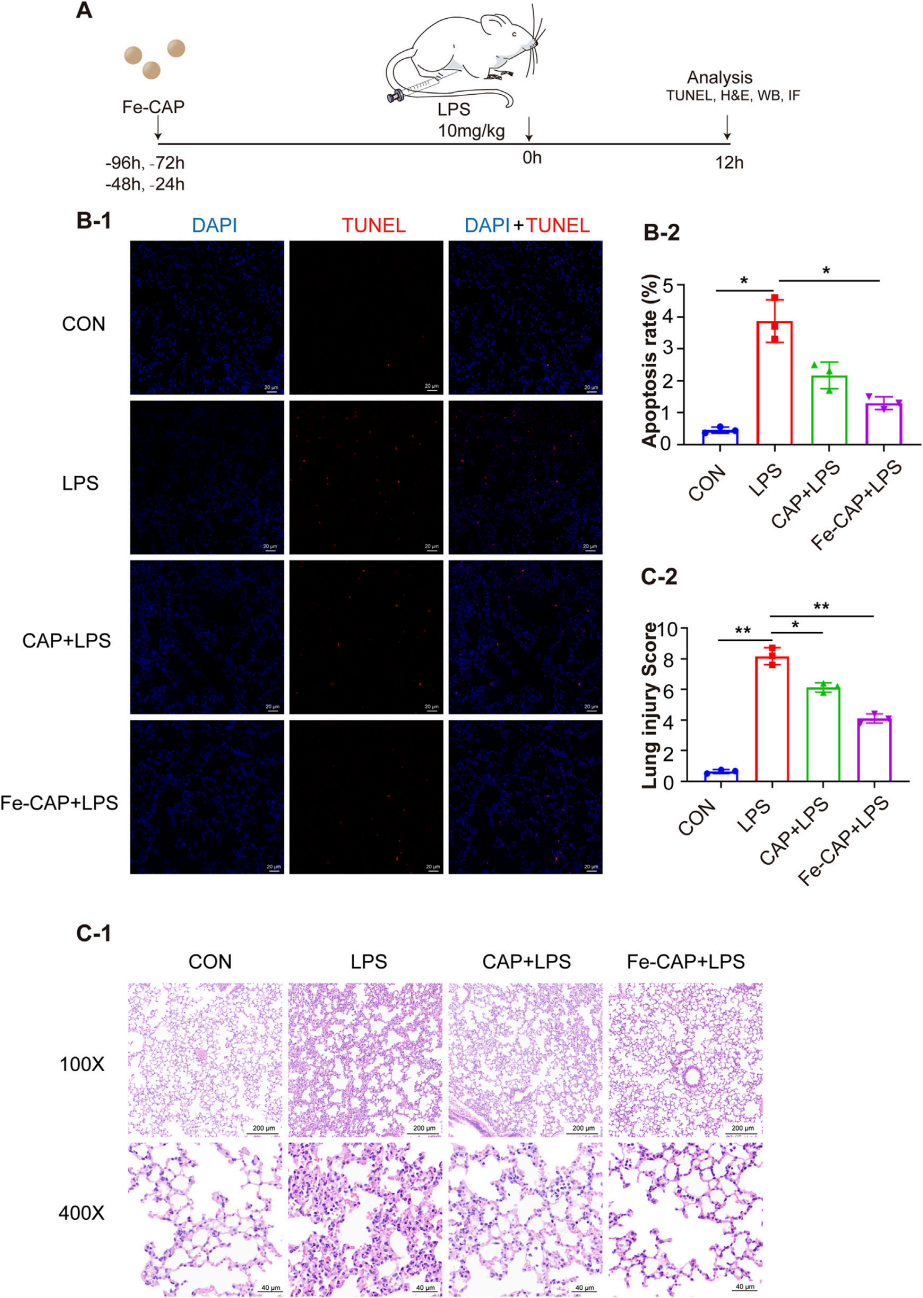
Fe-CAP NPs repaired the barrier structure and decreased apoptosis. Schematic illustration of the experiment conducted here **(A)**. The apoptosis of lung tissue was detected by TUNEL kit **(B-1, B-2)**. H&E staining **(C-1)** and lung injury score **(C-2)** of lung tissue.

The flow chart of animal experiments was shown in Figure 6A. Hematoxylin and eosin (H&E) staining was conducted on lung tissues of each group (Figures 6C–1). Lung sections revealed substantial pathological alterations in the LPS group, characterized by thickening of alveolar septum, interstitial hemorrhage, pronounced alveolar collapse, massive infiltration of interstitial lymphocytes and neutrophils, and high injury score (LIS) (Figures 6C–2). However, treatment with Fe-CAP lead to reduction in lung tissue inflammation and damage, surpassing the efficacy of CAP treatment.

TUNEL assay (Figure 6B) was used to evaluate the apoptosis rate in mouse lung tissue from different groups, which was increased by LPS. Nevertheless, pretreatment with Fe-CAP NPs mitigated the effects of LPS and notably reduced apoptosis.

### Fe-CAP NPs increased M2 macrophages in lung tissue of LPS-induced mouse

**FIGURE 7:**
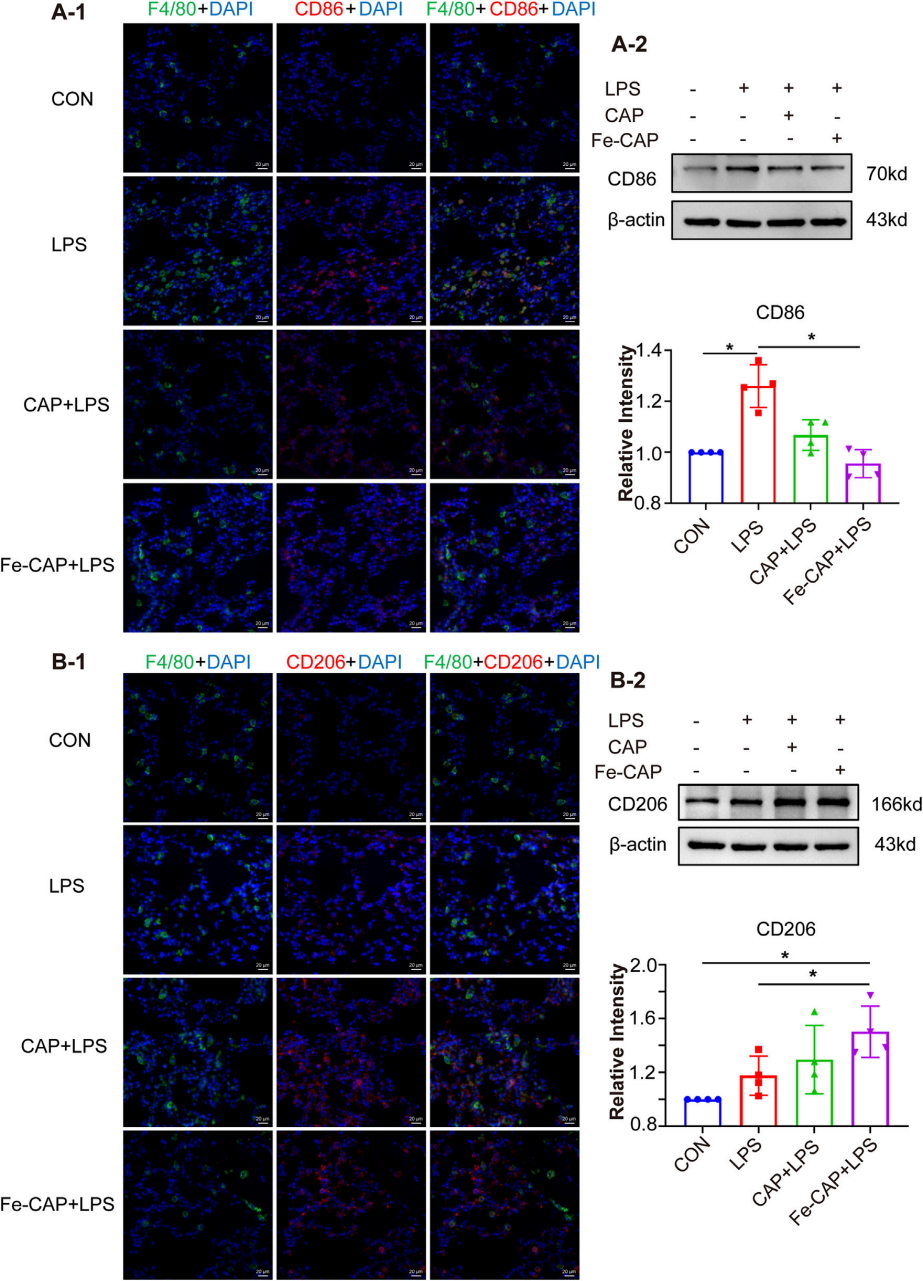
Polarization of macrophages in lung tissue. The expression of CD86 was detected by immunofluorescence **(A-1)** and Western blot **(A-2)**. The expression of CD206 was detected by immunofluorescence **(B-1)** and Western blot **(B-2)**.

Western Blot results indicated that the expression of CD206 (Figures 7B–2) in lung tissue of Fe-CAP NPs + LPS group increased compared with the LPS group. Notably, capsaicin alone also upregulated CD206 expression, but not as effectively as Fe-CAP NPs. Similarly, the expression of M1 macrophage marker CD86 (Figures 7A–2) was increased in LPS group and significantly decreased after Fe-CAP pretreatment.

The results of immunofluorescence indicated that Fe-CAP NPs effectively increased the fluorescence intensity of CD206 (Figures 7B–1) and decreased the fluorescence intensity of CD86 (Figures 7A–1).

## Discussion

Sepsis is caused by dysfunction of the host’s response to infection, with high morbidity and mortality (Druzak et al., 2023; Wang and Liu, 2023; Bruno et al., 2021). Timely detection and intervention in inflammatory response disorders may be a promising strategy to alleviate sepsis (Singer et al., 2016; Kapper et al., 2024; Olander et al., 2021). In our previous study, we successfully synthesized Fe-CAP NPs. The characterization of Fe-CAP NPs revealed that the nanozyme had favorable morphology and stability. We conducted a series of *in vivo* and *in vitro* experiments to prove that Fe-CAP NPs could suppress the expression of pro-inflammatory factors in macrophages, increase the expression of anti-inflammatory factors, and mitigate lung tissue damage, thereby reducing inflammation and alleviating lung injury in sepsis (Wang et al., 2024).

Macrophages play a crucial role in maintaining homeostasis, innate immunity, and mounting immune response to antigenic stimuli (Caiado and Mans, 2022; Loft et al., 2022; Yao et al., 2022). When inflammation occurs in the lungs, immune cells such as neutrophils are recruited to infiltrate the lung tissue (Su et al., 2023). Emerging evidence implicates macrophages are also involved in the pathogenesis of ALI/ARDS, including regulating inflammatory response and repairing damaged lung tissue (Huang et al., 2018; Johnston et al., 2012). During the initial phase of sepsis, pro-inflammatory cytokines such as interferon-γ (IFN-γ) and lipopolysaccharide (LPS) can instigate the polarization of M1 macrophages in the host. The increasing number of M1 macrophages will release an abundance of inflammatory cytokines, such as IL-6, tumor necrosis factor-α (TNF-α) and ROS, and further aggravate the inflammatory response (Murray and Wynn, 2011; Feng et al., 2014). On the contrary, in the later stages of sepsis, excessive increase of M2-type macrophages will secrete more anti-inflammatory factors IL-10, transforming growth factor-β (TGF-β), etc., contributing to immune suppression in the host (Feng et al., 2014; Murray and Wynn, 2011; Xu et al., 2019). The distinct polarization states of macrophages are pivotal facets involved in the regulation of immune homeostasis in the host micro-environment (Liu et al., 2014; Cheng et al., 2018), and also play a crucial role in the initiation and maintenance phases of tissue repair (Hang et al., 2024). Therefore, we want to explore whether Fe-CAP alleviates septic lung injury by modulating the polarization and function of macrophages.

CD86 and CD206 are phenotypic markers of M1 macrophages and M2 macrophages, respectively (Kigerl et al., 2009). In the present study, we stimulated BMDM with LPS and found that the expression of CD86 was notably increased. Interestingly, Fe-CAP NPs pretreatment significantly decreased the expression of CD86 but increased the expression of CD206. Jin et al. found that Koumine could alleviate sepsis-associated liver injury by inhibiting M1 polarization of macrophages and promoting M2 polarization (Jin et al., 2022). Liu et al. found that α-KG pretreatment could suppress M1 marker gene expression (TNF-α) and enhance M2 marker gene expression (Arg-1) to alleviate LPS-induced ALI/ARDS in mice (Lim et al., 2019). This is consistent with our findings. Interestingly, according to the experimental results, we found that the expression of CD206 increased after LPS stimulation of BMDM. This is consistent with the description in the literature, but this author focused more on the M1/M2 ratio after LPS stimulation (Wang et al., 2019). We consider because there are more detailed classifications of M2 macrophages, such as M2a, M2b, M2c, etc. M2a Macrophage is involved in immune and inflammatory responses, producing pro-inflammatory and anti-inflammatory cytokines (Li et al., 2023). Therefore, there will be a small amount of BMDM polarization to M2 type after LPS stimulation. These collective results indicate that Fe-CAP NPs exerts a regulatory influence on macrophage polarization in sepsis, inhibits the excessive activation of M1 macrophage and increases the proportion of M2 macrophage.

When the tissues of the body are damaged, macrophages will be recruited and migrate to the injured site, phagocytosis of degrading cell debris and invading microorganisms, and provide a favorable micro-environment for subsequent tissue repair (Kloc et al., 2018; Yang et al., 2021). But in severe sepsis, macrophages and inflammatory cells in the tissue are activated and recruited, further amplifying the inflammatory response (Delano and Ward, 2016; Bao et al., 2011). Excessive migration and phagocytosis by macrophages are known to potentially result in tissue harm and hinder recovery (Wang et al., 2021). Recent research has revealed that miR-25 inhibition of macrophage migration reduces the release of pro-inflammatory factors in sepsis (Zhu et al., 2018). The anti-inflammatory agent (Dp44 mT) notably reduced the phagocytosis ability of macrophages LPS treatment (Lim et al., 2021). Our investigation found similar results that Fe-CAP could reduce the excessive migration a nd phagocytosis of LPS-activated macrophages. Given the role of oxidative stress in ALI, we assessed ROS levels in different groups of BMDM. The results showed that Fe-CAP pretreatment could significantly suppressed ROS levels. Furthermore, we analyzed apoptosis-related proteins in distinct BMDM cell sets, indicating a significant increase in the pro-apoptotic protein BAX and cleaved caspase-3 levels, along with a decrease in the anti-apoptotic protein Bcl-2 following LPS exposure. Conversely, these effects were reversed in the Fe-CAP pretreatment group. A study had shown that Matrine can reduce myocardial damage induced by sepsis by inhibiting apoptosis and ROS (Xiao et al., 2023). It is similar to our results. The above results suggest that Fe-CAP NPs can not only regulate the polarization and function of macrophages, but also reduce ROS levels and apoptosis, suggesting the potential therapeutic prospect of Fe-CAP NPs in sepsis.

The JAK - STAT signaling pathways play critical roles in numerous biological processes, including cell proliferation, apoptosis, differentiation and immune regulation. When factors bind to their receptors, JAK is phosphorylated and activated, which subsequently leads to phosphorylation and dimerization of STAT (Banerjee et al., 2017). Kong et al. found that Hesperetin derivation-12 (hdd-12) regulates macrophage polarization by regulating JAK2/STAT3 signaling pathway (Kong et al., 2019). We used RNA sequencing to screen potential targets and signaling pathways for Fe-CAP regulation of macrophage polarization. Based on KEGG and GO analysis results, we selected the JAK2/STAT3 pathway for validation. Our findings demonstrated a significant increase in the levels of phosphorylated JAK2 and STAT3 following LPS stimulation of BMDM. Fe-CAP preconditioning can reverse this phenomenon, suggesting that Fe-CAP might influence macrophage polarization through the JAK2/STAT3 pathway.

In addition, we conducted further validation in mice. Specifically, in LPS-induced mice, Fe-CAP treatment effectively ameliorated histopathological changes and lung injury scores, reduced cell apoptosis, and increased the number of M2-type macrophages. This also confirms the results of our cell experiments.

In conclusion, our findings provide further theoretical support for Fe-CAP NPs to alleviate acute lung injury. Nonetheless, there are some limitations to this study. The mechanism of the anti-inflammatory effect of Fe-CAP NPs is not deeply studied, and gene knockout is needed for further verification.

## Conclusion

In summary, this study demonstrated that Fe-CAP NPs alleviate the septic lung injury by regulating macrophage polarization, function, and reducing apoptosis and ROS levels through *in vitro* and *in vivo* experiments.

## Data Availability

The original contributions presented in the study are included in the article/Supplementary Material, further inquiries can be directed to the corresponding authors.

## Abbreviations

ALI, Acute lung injury; LPS, Lipopolysaccharide; BMDM, Bone marrow-derived macrophages; Fe-CAP NPs, Iron-capsaicin-based nanoparticles; CAP, Capsaicin; NF-κB, Nuclear factor kappa-B; ARDS, Acute respiratory distress syndrome; PVP, Polyvinylpyrrolidone; i. p., Intraperitoneal; PBS, Phosphate-Bufer Saline; DMEM, Dulbecco’s Modifed Eagle Medium; FBS, Fetal Bovine Serum; M-CSF, Macrophage colony stimulating factor; CCK-8, Cell Counting Kit 8; BCA, Bicinchonininc acid; LIS, The Lung Injury Score; H&E, hematoxylin and eosin; IFN-γ, interferon-γ; TNF-α, Tumor Necrosis Factor-α.
