# Supplementary figures and images for: Fe-capsaicin nanozyme attenuates sepsis-induced acute lung injury by regulating the functions of macrophages
Source: Front Bioeng Biotechnol. 2024 Nov 18;12:1509136. doi: 10.3389/fbioe.2024.1509136 (PMC11608995; doi:10.3389/fbioe.2024.1509136)

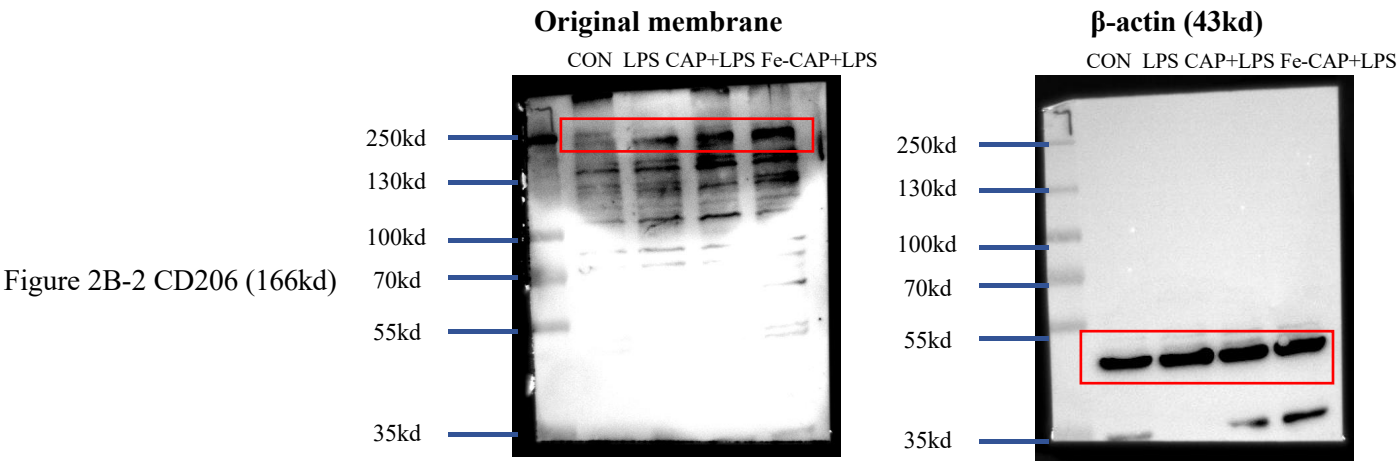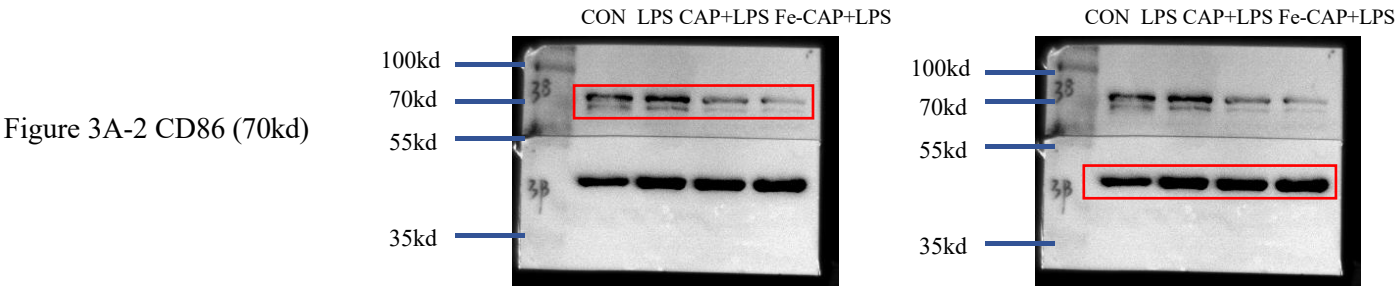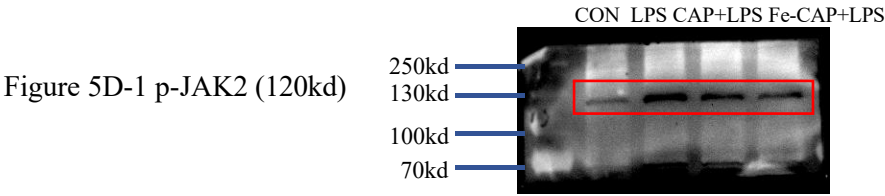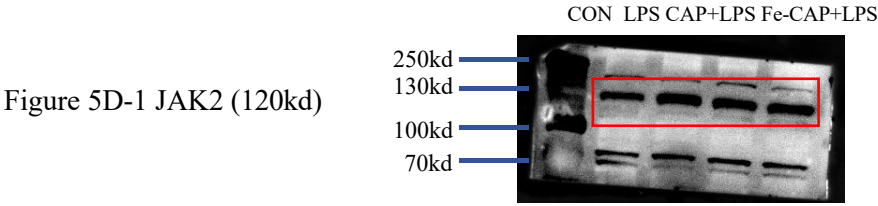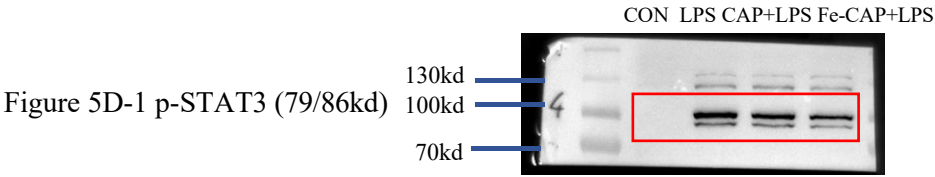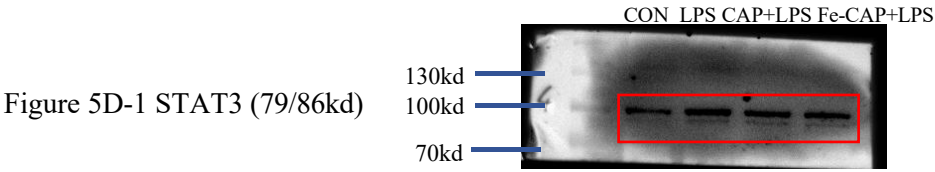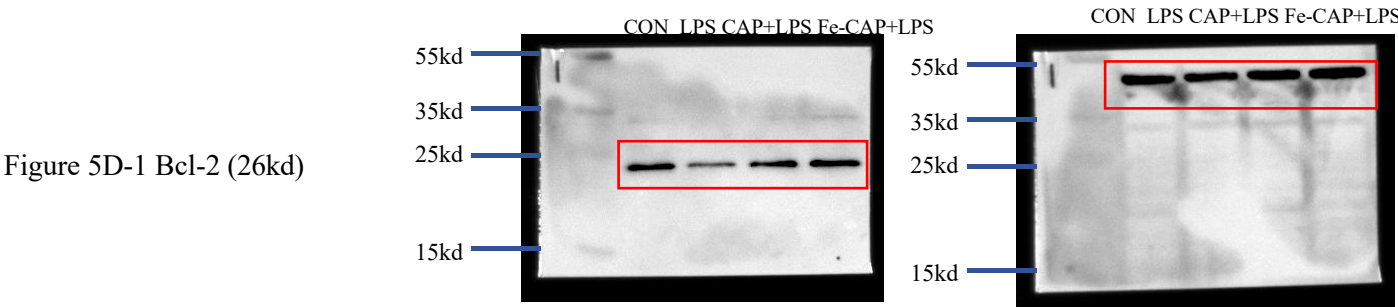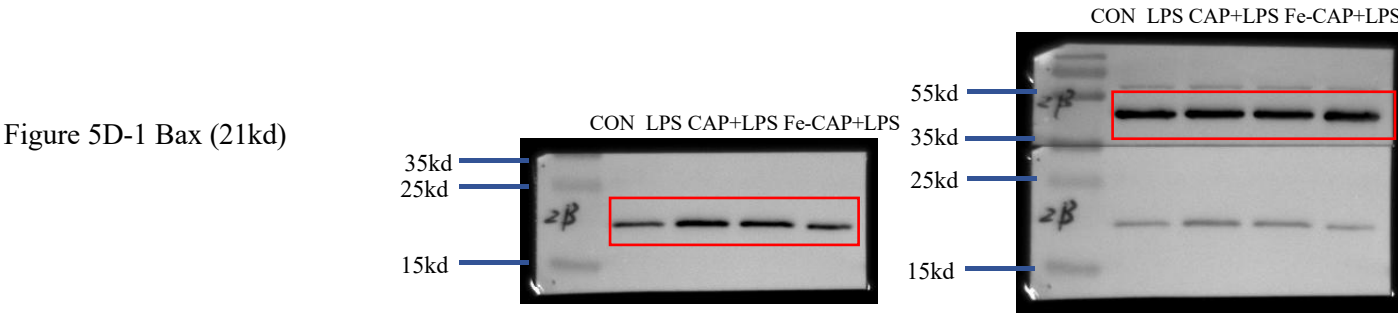

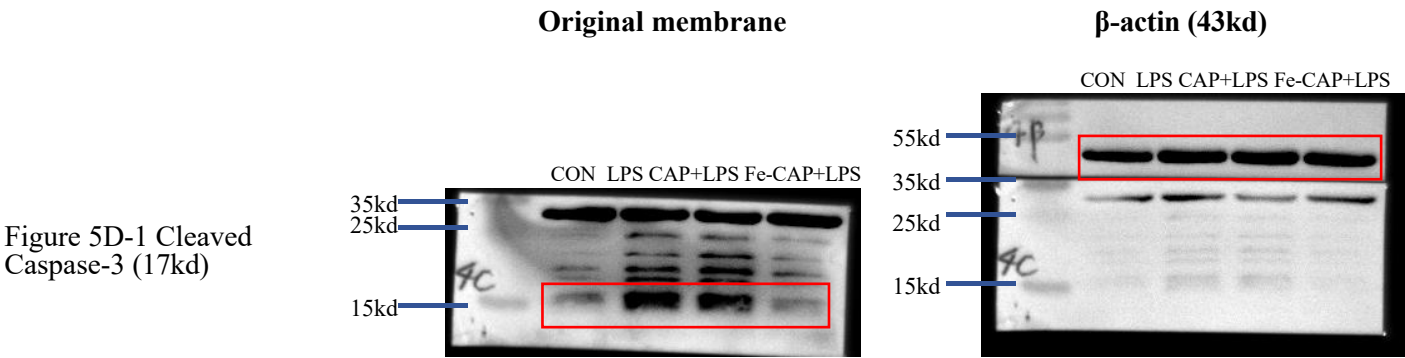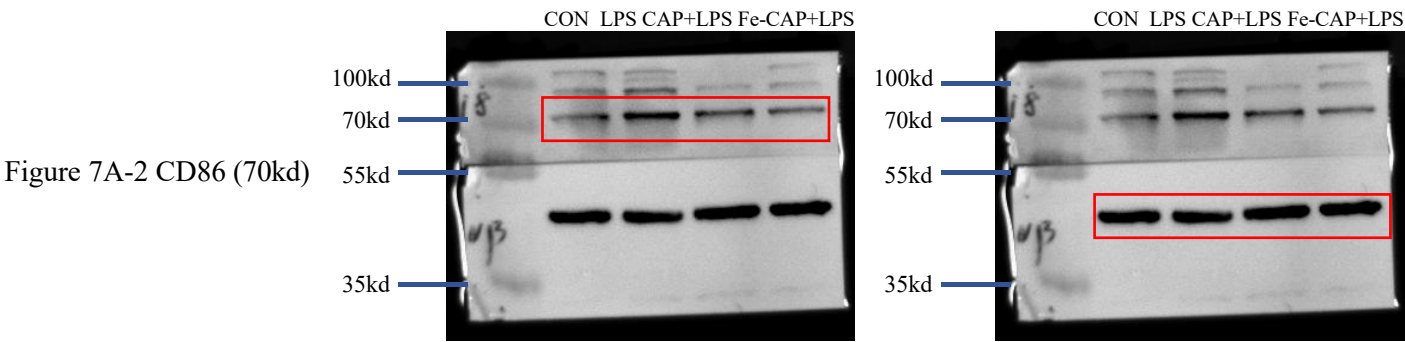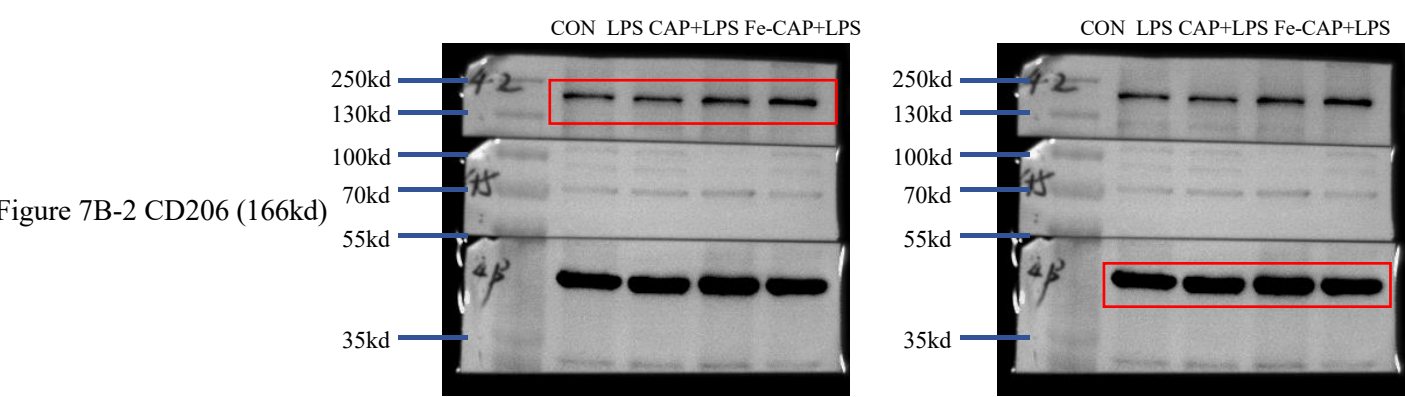

Supplement: Supplementary file 1 [file DataSheet1.PDF]
